# Pacemaker-Mediated Tachycardia: A Case Report

**DOI:** 10.7759/cureus.26583

**Published:** 2022-07-05

**Authors:** Rajwinder Gill, Vineet Meghrajani

**Affiliations:** 1 Department of Internal Medicine, Icahn School of Medicine at Mount Sinai Beth Israel, New York, USA; 2 Department of Cardiovascular Medicine, Icahn School of Medicine at Mount Sinai Beth Israel, New York, USA

**Keywords:** pacemaker mediated tachycardia, inappropriate rate responsiveness, sensor defect, tachycardia, minute ventilation sensor defect, pacemaker driven tachycardia

## Abstract

Pacemakers respond to the physical activity of patients by increasing the heart rate to compensate for the increased demand. They sense the physical activity of the patient with the help of different types of sensors that work with different mechanisms. In this case, we are presenting a 74-year-old male who was experiencing intermittent chest pain and palpitations. Following interrogation, he was found to have atrial paced tachycardias due to a tracking of the atrial tachycardia.

## Introduction

A pacemaker is a type of cardiac implantable electronic device that can generate electrical impulses. They can be temporary or permanent. The main indications for pacemaker placement are symptomatic bradycardia from the sinus node or AV node, second or third-degree heart block, long QT syndrome, recurrent syncope, etc. [1]. The pacemaker can be single chamber, dual chamber, and biventricular. Mode of pacemaker is specified by coding in which the first letter stands for the area being paced, the second letter for the area being sensed, the third letter states response of pacemaker, the fourth letter is for presence or absence of rate modulation, and the fifth letter specifies location or absence of multisite pacing [2].

## Case presentation

A 74-year-old male presented with intermittent chest pain. Chest pain was dull, substernal, and non-radiating. The pain started while walking and was relieved with rest. It was associated with shortness of breath, palpitations, and dizziness without syncope. He denied orthopnea, paroxysmal nocturnal dyspnea, cough, and leg swelling. He had a history of atrial fibrillation status post cardioversion and ablation, coronary artery disease, diabetes mellitus, hypertension, carotid artery stenosis status post endarterectomy, and sick sinus syndrome status post pacemaker implantation.

**Figure 1:**
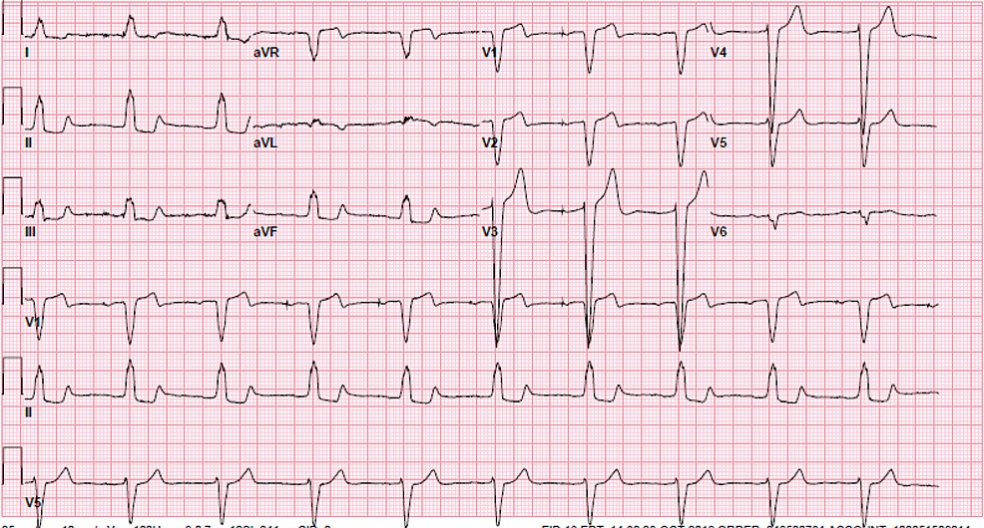
Initial EKG showing sinus rhythm with first degree AV block and left bundle branch block

**Figure 2:**
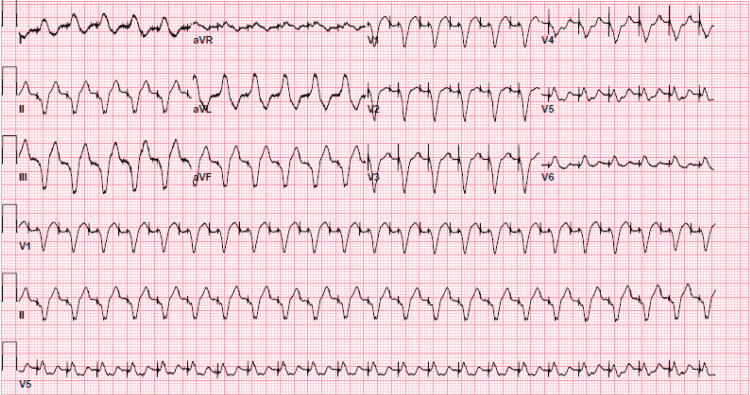
Electrocardiogram (EKG) showing atrial-paced tachycardia with rate of 139 beats/minute

A physical exam revealed a heart rate of 74 beats per minute, a temperature of 37 degrees Celsius, a respiratory rate of 16 per minute, and oxygen saturation of 97% on room air. The cardiovascular exam was unremarkable. Initial EKG was pertinent for sinus rhythm with first-degree atrioventricular (AV) block and left bundle branch block as shown in Figure 1. He was admitted to the cardiac care unit. Telemetry showed multiple events with dual-paced tachycardia to 140 beats per minute. He began to have chest pain when tachycardic. EKG at that time showed dual-paced tachycardia with a rate of 139 as shown in Figure 2. The initial laboratory workup was unremarkable as shown in Table 1.

**Table 1:** Initial laboratory work-up

Test	Results	Reference Range
White Blood Count (WBC)	6.5	4.5-11.00 k/uL
Hemoglobin	13	13.6-16.3 g/dL
Platelet	244	150-450 k/uL
Sodium	139	135-145 meq/L
Potassium	4.2	3.5-5.2 mmol/L
Chloride	111	96-108 mmol/L
Phosphorus	3.6	2.4-4.7 mg/dL
Magnesium	2.1	1.5-2.5 mg/dL
Creatinine	0.68	0.5-1.1 mg/dL
Blood Urea Nitrogen	16	6-23 mg/dL
Brain Natriuretic Peptide (BNP)	156	0.0-100 pg/mL
Troponin	<0.010	<0.031 mg/dL
Aspartate aminotransferase	26	1-35 U/L
Alanine aminotransferase	28	1-45 U/L
Alkaline phosphatase	56	38-126 U/L

**Figure 3:**
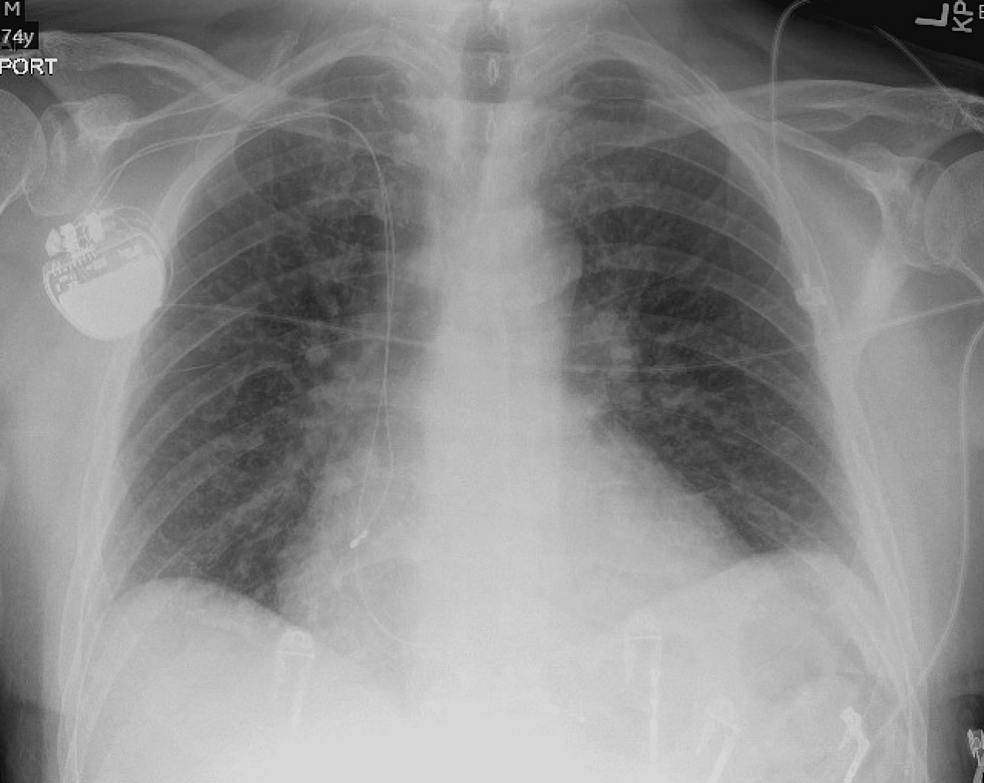
Chest X-ray showing dual-chamber pacemaker with intact leads

Device interrogation was done and it showed atrial-paced tachycardia and multiple short paroxysms of atrial-paced tachycardia due to tracking of atrial tachycardia. There was no evidence of atrial fibrillation on device interrogation. The device was reprogrammed from DDDR (dual, dual, dual, rate-responsive) mode to DDI (dual, dual, Inhibition) mode. Rate adaptiveness was turned off. The minute ventilation sensor was deactivated and the device was reprogrammed for a motion-based accelerometer only. Beta-blocker dose was increased as tolerated. On telemetry after changing the settings, he didn’t get any episodes of atrial tachycardia. Coronary angiography was done given the nature of the chest pain which was found to have non-obstructive mild two-vessel coronary artery disease. Chest X-ray showed the intact placement of pacemaker leads without any fracture of the leads shown in Figure 3. On outpatient follow-up, the patient didn't have these episodes and remained in sinus rhythm for four months. Atrial fibrillation came back after that which was treated with a second ablation following which he remained in sinus rhythm.

## Discussion

Pacemakers have two types of sensors to adjust the heart rate according to the physical activity of the patient. The first one is the activity sensor which is a piezoelectric accelerometer that responds to the acceleration/motion and produces the electric charge in proportion to acceleration. The change in voltage is used to estimate the physical activity based on the algorithms. The second type of sensor is the minute ventilation sensor. These sensors can measure the impedance of the thoracic cavity. During the respiratory cycle, impedance changes due to the change in air content of the thoracic cavity. This change in impedance helps these sensors to estimate the respiratory rate and minute ventilation further and pacemaker changes the rate of heart pacing based on activity sensing by these sensors [3].

Current literature states that dual-sensing has a more physiological response to physical activity and patients have a better quality of living rather than each one of the single sensors [4-5]. Some patients with an accelerometer as the only sensor of physical activity complain of fatigue during the exercise as heart rate does not increase as needed. There is an ongoing study for comparing the accelerometer and minute ventilation sensors.

Pacemaker-mediated tachycardia (PMT) could be due to multiple mechanisms like tracking of sinus tachycardia or atrial arrhythmias, tracking of electromagnetic devices, and endless loop tachycardia due to a reentrant circuit, etc. Endless loop tachycardia is the most common mechanism of pacemaker-mediated tachycardia. The usual symptoms of PMT are chest pain, palpitations, syncope, or rarely heart failure symptoms. Temporary treatment can be done by placing the magnet on the pacemaker pocket but definitive management requires reprogramming of the device [6-7].

Sensor-mediated tachycardia can be due to false sensing by minute ventilation sensors resulting in a false estimation of minute ventilation due to a few reasons. The concurrent use of other monitoring devices like cardiac monitoring equipment, electrocardiograph equipment, external defibrillators, and respiratory monitoring equipment can lead to incorrect interpretation as elevated minute ventilation by minute ventilation sensor and lead to pacemaker-induced tachycardia [8-9]. Electrocautery can also lead to false estimation of minute ventilation by the sensors and lead to tachycardia [10-11]. Sensor-mediated tachycardia can also be due to tracking the atrial tachycardia or arrhythmias [6-7].

Patients admitted to an inpatient unit for cardiac monitoring should have the rate-responsive mode turned off. In our case, patient symptoms improved after turning off the minute ventilation sensor. His intermittent chest pain, palpitations, and dizziness were most likely due to atrial-paced tachycardia.

## Conclusions

Pacemaker-mediated tachycardia due to tracking of atrial rate by a sensor is an uncommon mechanism. Atrial ectopy is common after ablation in patients with atrial fibrillation. In this case, tachycardia resolved after reprogramming the device and increasing the dose of beta blocker to suppress the atrial ectopy. Although atrial tachycardia while on telemetry could be due to interference, tracking atrial ectopy would explain the symptoms of this patient in the field. Chest pain of the patient is likely related to increased demand during tachycardia.
